# An Integrated Method for Tracking and Monitoring Stomata Dynamics from Microscope Videos

**DOI:** 10.34133/2021/9835961

**Published:** 2021-04-09

**Authors:** Zhuangzhuang Sun, Yunlin Song, Qing Li, Jian Cai, Xiao Wang, Qin Zhou, Mei Huang, Dong Jiang

**Affiliations:** Regional Technique Innovation Center for Wheat Production, Ministry of Agriculture, Key Laboratory of Crop Physiology and Ecology in Southern China, Ministry of Agriculture, Nanjing Agricultural University, Nanjing 210095, China

## Abstract

Patchy stomata are a common and characteristic phenomenon in plants. Understanding and studying the regulation mechanism of patchy stomata are of great significance to further supplement and improve the stomatal theory. Currently, the common methods for stomatal behavior observation are based on static images, which makes it difficult to reflect dynamic changes of stomata. With the rapid development of portable microscopes and computer vision algorithms, it brings new chances for stomatal movement observation. In this study, a stomatal behavior observation system (SBOS) was proposed for real-time observation and automatic analysis of each single stoma in wheat leaf using object tracking and semantic segmentation methods. The SBOS includes two modules: the real-time observation module and the automatic analysis module. The real-time observation module can shoot videos of stomatal dynamic changes. In the automatic analysis module, object tracking locates every single stoma accurately to obtain stomatal pictures arranged in time-series; semantic segmentation can precisely quantify the stomatal opening area (SOA), with a mean pixel accuracy (MPA) of 0.8305 and a mean intersection over union (MIoU) of 0.5590 in the testing set. Moreover, we designed a graphical user interface (GUI) so that researchers could use this automatic analysis module smoothly. To verify the performance of the SBOS, the dynamic changes of stomata were observed and analyzed under chilling. Finally, we analyzed the correlation between gas exchange and SOA under drought stress, and the correlation coefficients between mean SOA and net photosynthetic rate (Pn), intercellular CO_2_ concentration (Ci), stomatal conductance (Gs), and transpiration rate (Tr) are 0.93, 0.96, 0.96, and 0.97.

## 1. Introduction

Stoma is one of the most important apparatuses controlling gas exchange and water transpiration between plants and the atmosphere, to closely coordinate with photosynthesis and water or nutrient uptake [1, 2]. The number and behavior of stomatal apertures partly reflect the metabolic level and water status of plants [3]. However, due to the limitation of observation tools in the early years, most researchers have tacitly presumed that response of a single stoma to environmental stimulus was independent and common with minor random variation [4]. In this context, researchers only observed the behavior of several single stomata to prove the stomatal responses to environments. However, recent evidence suggested that stomatal behavior frequently exhibited dramatic spatial heterogeneity in the same leaf [5]. In some extreme cases, two adjacent stomata could behave reversely with one in open and another in close. This phenomenon is the so-called patchy stomata [6].

Patchy stomata have been evaluated in many plant species under a series of environmental adversities in recent years [7]. For example, the patchy stomatal closure could induce the heterogenic photosynthesis of the Mediterranean holm oak (*Quercus ilex* L.) leaf under severe drought conditions [8]. In some tropical tree species, the patchy stomata could open in several scales within the same leaf, which induces depressed leaf CO_2_ exchange [9]. The changing CO_2_ assimilation patterns were observed to be consistent with the performance of patchy stomata in bean leaf [10]. Interestingly, the temporally asymmetric response of stomatal conductance could confer maximized photosynthesis per unit water loss to cope with heat stress in wheat [11]. Therefore, it is important to clear the heterogeneous behavior of stomata in response to varied environmental conditions. However, the lack of time-course and real-time observation method limits the better understanding of this regard.

The stomatal observation is the premise of understanding patchy stomata, and there are indirect and direct ways to observe stomatal morphology and movement. The indirect methods are usually nonimage methods by evaluating stomatal conductance according to the exchange rate of water vapor and CO_2_ via the stomatal aperture. The direct methods are usually image-based observation and analysis on the thermography [12] and fluorescence images [4], or the recently reported plant surface impression technique using polyaddition silicone rubber [13]. Although the direct image-based methods are visualized, they have many unstable factors affected by the environment. For instance, high atmospheric humidity dramatically decreases the correlation between stomatal conductance and temperature, and the heat flow further causes blurred image boundaries, which limits the adoption of the thermography method in stoma behavior observation [12]. The same problem also occurs in the fluorescence images method [8]. The silicone rubber impression technique performs a more precise analysis of stomata aperture morphology and allows observation of the temporal heterogeneous distribution of stomata in combination of the scanning electron microscopy [14]. However, the specimen preparation is complicated and time consuming and is an invasive method which could not observe the time-course dynamic responses of stomata to environmental conditions. In another word, the above-mentioned methods are far than enough for the observation of the patchy stomatal behavior. Recently, we developed a method that can automatically identify and count stomata with a low-cost portable microscope [15]. Our method provides a potential approach to observe the time-course dynamics of the stomatal aperture movement. Real-time observation is conducive to better reflect the stomata response to external factors, but it puts forward higher requirements for feature extraction.

The other focus of studies is to extract stomata features from pictures. Most scholars measure stomata pores manually using ImageJ, which is time and labor consuming. With the development of technology, the machine learning (ML) and deep learning (DL) are widely used in the analysis of stomatal morphology, which is efficient and accurate. Jayakody et al. proposed an automatic method for pore measurement of grape varieties with machine learning and histogram of oriented gradients (HOG) [16]. Li et al. used a faster region-based convolutional neural network (Faster R-CNN) to segment single stoma from different plant images and then measured these single stomatal morphological features with a manual CV model [17]. Song et al. obtained the contour coordinates of the pore regions in microscope images of leaves through Mask R-CNN (region-based convolutional neural network), which is an instance segmentation model [18]. Although these methods can obtain stomatal morphological features automatically or semiautomatically, detection dataset in these methods is static images, which cannot reflect dynamic changes of stomata. Actually, the stomata are very sensitive and usually quickly tend to close to environmental variations [19]. A noninvasive, real-time, and on-site system for stomatal aperture movement observation and analysis is required to investigate the time-course dynamic stomata behavior and to better understand the significance of the patchy stomata.

In this study, we further develop a new system for observing stomata behavior to facilitate the study of patchy stomata of plants. Specifically, we make the following works:

1. We fully utilized portable microscopes to shoot the videos of stomatal movement in a real-time and noninvasive way.
2. The system can automatically quantify the changes of stomatal apertures in stomatal videos through object tracking and semantic segmentation. Further, we designed a graphical user interface (GUI) so that researchers can use this automatic analysis module smoothly.
3. We successfully observed and analyzed the phenomenon of patchy stomata under chilling and drought stress.

## 2. Methods

**Figure 1:**
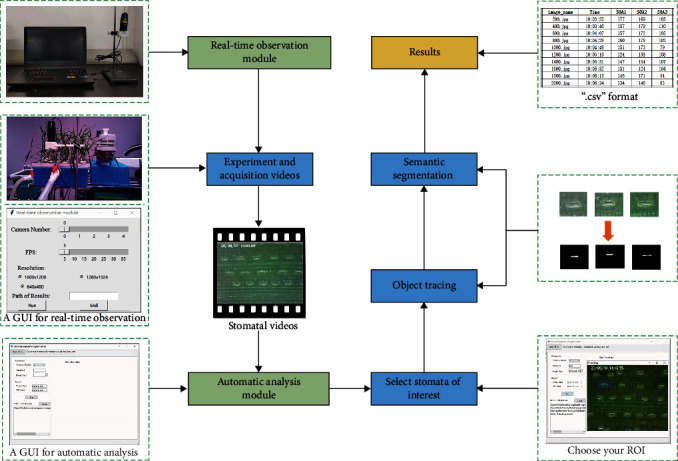
Workflow of SBOS.

The stomatal behavior observation system (SBOS) is a dynamic stomatal observation and analysis system. The whole system includes two modules: the real-time observation module and the automatic analysis module (Figure 1). Firstly, we shot videos of stomatal dynamic changes using the real-time observation module. Secondly, stomata were selected randomly to obtain time-series stomatal images by the object tracking, and then time-series stomatal images were put into the semantic segmentation to calculate pixel number of stomatal aperture (the so-called stomatal opening area (SOA)).

### 2.1. The Real-Time Observation Module

The real-time observation module included a portable microscopy (Anyty portable microscopy 3R-MSUSB601), a platform for both fixation and operation of the microscope, and a personal laptop. The real-time observation module was showed in the upper left corner of Figure 1. The portable microscope was connected to the laptop via USB cable, and a platform fixed both the microscope and the plant leaf. This platform was produced by 3D printing, and a 3D model was provided in the supplemental material S5. Finally, the laptop controlled the portable microscope for video capture by a graphical user interface (GUI) (Python version, https://github.com/shem123456/Stomata-segmentation-with-GUI). Compared to our previous work in Sun et al., the real-time observation module was characterized by the following new features. (1) The module was easily operated with the aid of a GUI. (2) The video could be saved as the format of “∗.avi”, and the time and date were marked in upper left corner of the video frame. (3) A laptop could control multiple portable microscopes simultaneously.

### 2.2. The Automatic Analysis Module

After shooting videos, the automatic analysis module was used for data extraction. The automatic analysis module integrates both object tracking and semantic segmentation. Further, we packaged this module into an executable software and designed a graphical user interface (GUI) for users without any knowledge of programming. The details of each step are introduced in the following paragraphs.

#### 2.2.1. Object Tracking

As one basic task of computer vision, the object tracking continuously predicts the size and position of the targets in the time-series video frames. It is a very easy task for object tracking in our cases. Because the stomata are immovable while only vary in opening size during observation, a KCF (Kernelized Correlation Filter) object tracker was adopted instead of the deep learning-based trackers that need to be trained with lots of samples. The KCF method is to train a correlation filter based on the information of the current and previous frame and then calculate the correlation with the newly input frame. The confidence graph obtained is the predicted tracking result [20]. Here, a built-in OpenCV object tracking module was invoked to create three KCF object trackers for tracking multiple stomatal targets. Once the targets were labeled in the initial frame, the tracker could automatically identify and localize all stomata in the subsequent frames.

#### 2.2.2. Semantic Segmentation

**Figure 2:**
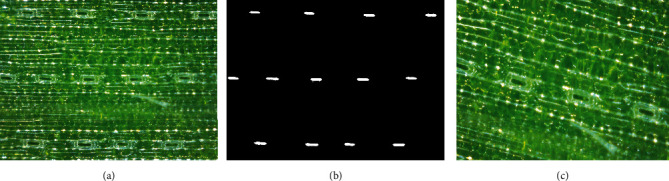
Images from training set: (a) an original image; (b) a mask image; and (c) an image with data augment.

*(1) Images Acquisition and Labeling*. To train a deep learning model, it usually needs a large number of images. Therefore, many stomatal observation videos were obtained using the shooting module, including different growth stages, such as the three-leaf stage and mature stage, as well as treatments with drought, low-temperature, and high-temperature. Then, we wrote the code in Python that can capture an image from the video every 1000 frames. At the same time, images of different scales were cropped out for diversity in the training set (Figure 2(a)). 420 images were annotated after selection. Finally, the dataset was available online at https://github.com/shem123456/stomata-segmantic-segmentation.

A JavaScript image annotation tool based on image segmentation was used [21]. Two categories were annotated: background and stomatal aperture. The RGB value of background is [0, 0, 0], and the RGB value of stomatal aperture is [1, 1, 1]. The RGB value of the stomatal aperture is close to the background, so the two categories could not be distinguished by the naked eye in the mask image. For visualization, we multiplied the pixel value of the mask image by 255 to get a visual mask image (Figure 2(b)).

Data augmentation was used in order to obtain a robust model. Many studies have shown that data augmentation can improve the performance of the model, such as rotation, flipping, and scaling [22]. Therefore, the Augmentor tool was used to expand the training set [23], and the following measures were adopted: (1) Images were rotated, and the maximum rotation angle between left and right was 25°, which were randomly executed according to a probability of 80%. (2) Images were flipped left and right, which were randomly executed according to a probability of 50%. (3) Images were scaled down to 0.85 times of the original, which were randomly executed according to a probability of 30%. After data augmentation, we added 755 more images (Figure 2(c)).

**Figure 3:**
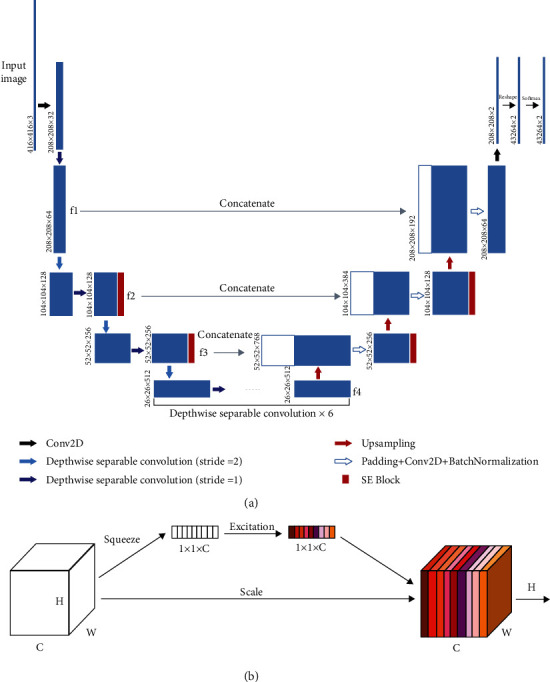
Network architecture: (a) UNet network; (b) a channel attention mechanism.

*(2) Network Architecture*. The UNet network was adopted, which is an encoder-decoder network structure. The encoder on the left is a normal feature extraction process, and the decoder on the right performs image upsampling and feature fusion (Figure 3(a)).

In the encoder, MobileNet was adopted as the backbone for feature extraction. Depthwise separable convolution is the basic unit of MobileNet, and it can decompose two smaller parts: depthwise convolution and pointwise convolution. Depthwise convolution uses different convolution kernels for each input channel. That is to say, a convolution kernel corresponds to an input channel. Pointwise convolution is actually a conventional convolution, but its convolution kernel size is 1 × 1. For a depthwise separable convolution, depthwise convolution is first used, and then pointwise convolution is used to combine the above output. In this way, the effect of depthwise separable convolution is similar to that of a standard convolution, but the advantage is that it will greatly reduce the amount of computation and the model parameters. In the encoder, MobileNet is used to extract the features from the original image, and four intermediate layers are obtained, named f1, f2, f3, and f4. In the decoder, new features are obtained by upsampling the f4 features, and then new features are concatenated with f3 after a convolution operation. Then, three upsampling operations are carried out, and finally, the features are softmaxed to get the segmentation output.

Attention mechanism is applied to the network structure. It is an interesting module, and it is inspired by the human vision. When humans scan an image, their vision will focus on the target area, and then they invest more attention in this area to obtain more detailed information and suppress other useless information. Therefore, the attention mechanism in deep learning is essentially similar to human vision, and the core goal is to select the information that is more critical to the current task. In this paper, the channel attention mechanism is adopted. Firstly, the feature is squeezed into a size of 1 × 1 to obtain the global feature at the channel level. Secondly, the global feature is excited to get the weight of different channels. Finally, the original feature is multiplied by the weight to scale feature channels (Figure 3(b)). This module is also called the Squeeze-and-Excitation block (SE block) [24]. This attention mechanism allows the model to pay more attention to the more important channels, while suppressing the unimportant channels [25]. Besides, the SE block is universal and can be embedded in any network architecture, so we embed 4 SE blocks in the original UNet network (Figure 3(a)).

*(3) Training*. Transfer learning was used [26], and all layers were initialized as in He et al. All models were trained on a high-performance computer with Intel i7 8700 central processing unit (CPU), 32 GB of memory, and the NVIDIA 2080 GeForce graphics processing unit (GPU). The operating system was Windows, and the network structure was constructed by Keras. The batch size was 5, and optimization was the Adam method with a learning rate of 0.001.

*(4) Evaluation Metrics of Semantic Segmentation*. In order to evaluate the performance of the model, pixel accuracy (PA), CPA (class pixel accuracy), MPA (mean pixel accuracy), and MIoU (mean intersection over union) are chosen. PA is the ratio of the correct pixel count to the total pixel count. CPA is the ratio of the correct total number of pixels per class to the total number of pixels per class. MPA is the mean value of CPA. IoU (intersection over union) is the ratio of intersection to union of the ground truth and prediction of each class. MIoU is the mean value of IoU.

#### 2.2.3. Graphical User Interface (GUI)

**Figure 4:**
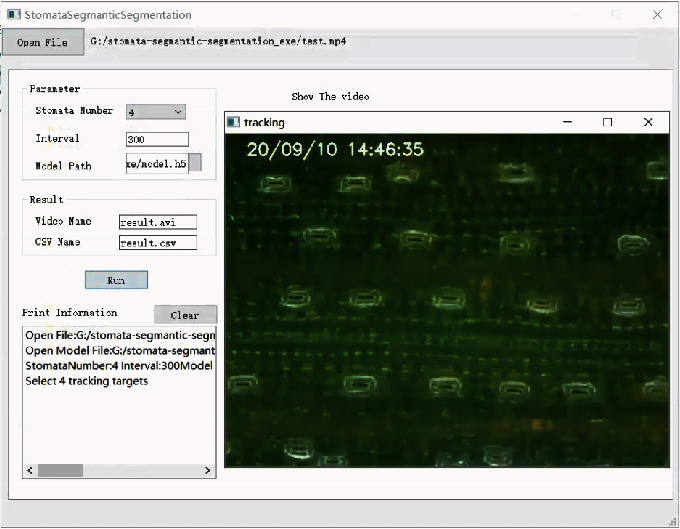
Graphical user interface (GUI).

In order to use this automatic analysis module smoothly, we packaged this module into an executable software and designed a graphical user interface (GUI) (Figure 4). This graphical user interface, which could run directly on any Windows system, was written using Pyqt5 (a python library) and packaged by PyInstaller (a python library). The entire software operation process only needs four steps. (1) Click on “Open File” to select a video file; (2) set up the parameters which includes stomata number, interval, and model path; (3) modify the path and name of the results; and (4) click on “Run” and draw rectangles on the area of interest. Finally, the result of SOA was stored in “.csv” format files which can be exported easily for analysis by other software. In the supplemental material S1, we also provided a full manual to guide users how to use it.

## 3. Experiments

### 3.1. Experiment I: Performance of Different Semantic Segmentation Models on the Test Set

We trained four semantic segmentation models in all. A model did not use data augmentation and SE blocks. The B model used data augmentation without SE blocks. The C model used SE blocks without data augmentation. The D model used both data augmentation and SE blocks. As mentioned in “The Automatic Analysis Module,” the remaining 78 samples were used for testing the performance of these models. Testing samples were normalized and resized into the size of 416 × 416 before they were put into the neural network. The test program used the same environment to train data. We calculated the evaluation metrics of PA, CPA, MPA, and MIoU after obtaining the mask images.

### 3.2. Experiment II: Changes of Stomatal Behaviors during Chilling Stress in Wheat

To verify the performance of the SBOS in different environments, we designed an experiment in wheat chilling stress. Uniform seeds of winter wheat Yangmai 16 (*Triticum aestivum* L.) were selected, and surface sterilized with 15% H_2_O_2_ for 10 minutes and then rinsed several times with distilled water. The seeds were placed in quartz sand to germinate, and unanimous seedlings were selected and transplanted to plastic containers (45 cm in length, 35 cm in width, and 18 cm in height) for hydroponic cultivation, and the Hoagland nutrient solution was exchanged every three days. The temperature of the climate chamber was set at 22°C/18°C (day/night), with a 14 hours' photoperiod at 350 *μ*mol m^−2^ s^−1^.

During the four-leaf stage, the latest fully expanded leaf was fixed by fixation platform in shoot module. And then, the python program was used to open portable microscope. The image captured would show on the laptop. The magnification of the microscope was adjusted slightly until the stomata was observed clearly. Next, we adjusted the temperature of the climate chamber from 20°C to 2°C. The video of stomatal dynamic changes under chilling stress would be saved. The video had a resolution of 640 × 480 and a frame rate of 20 frame per second.

### 3.3. Experiment III: Changes of Stomatal Behaviors during Drought Stress in Wheat

**Figure 5:**
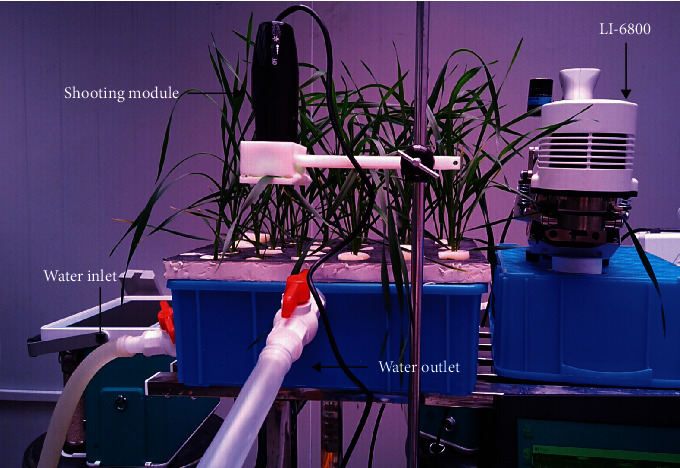
An experiment in wheat drought stress.

To verify the correlation between SOA and gas exchange, we designed an experiment in wheat drought stress. Yangmai 16 was planted for hydroponic cultivation in the climate chamber. The temperature of the climate chamber and the circadian rhythm were same as the Experiment II. To observe the dynamic changes of stomata under drought condition, drought stress was applied with the Hoagland nutrient solution which contains 15% PEG 6000 at the four-leaf stage. To avoid unexpected effects, a micro water circulation system controlled by a pump was designed to perform drought stress induced by the PEG 6000. Two pipes linked the plastic container and bucket, and the solution between these containers would be pushed and mixed by a water pump (Figure 5).

After the solution was fully mixed in the circulatory system, the latest fully expanded leaf was used to measure leaf gas exchange with a portable photosynthesis system (LI-6800, LI-COR Inc., USA), at a CO_2_ concentration of 400 *μ*mol mol^−1^ and a light level of 360 *μ*mol m^−2^ s^−1^, equal with the light intensity of the growing environment. The time was recorded by the auto log function in the above device, 40 minutes totally, with a 30-second interval. Meanwhile, the shooting module was used for real-time monitoring of the stomata in another latest fully expanded leaf synchronously. There were two biological replicates for each treatment.

## 4. Results

### 4.1. Performance of Semantic Segmentation Module

The result showed that it was beneficial to improve the model performance when data augmentation and SE blocks were used together. Four models were trained according to data augmentation and SE blocks. PA, CPA, MPA, and MIoU were used to evaluate test set for each model. Semantic segmentation models performed well on the test set (Table 1). Meanwhile, both data augmentation and SE blocks could improve the performance of model. For MIoU, data augmentation increased by 5.5% and 5.2%, and SE blocks increased by 1.1% and 0.8%. For MPA, data augmentation can increase by 2.2% and 6.2%, and SE blocks increased by -0.3% and 0.03%.

### 4.2. Verification of the SBOS under Chilling Stress

**Figure 6:**
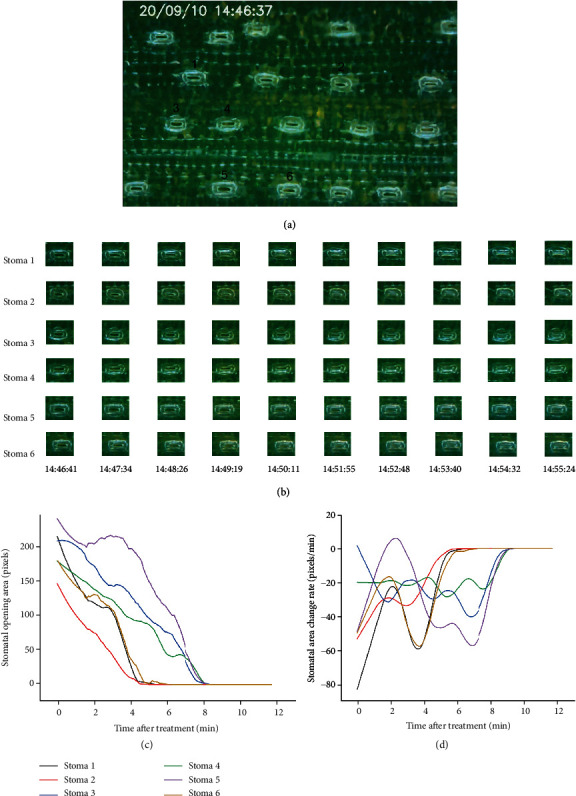
The dynamic changes of stomata under chilling stress. (a) The spatial distribution of stomata. (b) The result of object tracking. (c) The curve of SOA overtime. (d) The curve of stomatal area change rate.

We shot the whole process of stomatal change in wheat under chilling stress, and the relative video could be watched from Supplementary 3. From the video, we chose 6 stomata to calculate SOA in the video, and the spatial distribution of stomata is shown in Figure 6(a). Through the object tracking, stomatal pictures arranged in time-series were obtained (Figure 6(b)), and the result suggested the object tracking could locate every single stoma accurately. The time-series pictures were put into the semantic segmentation module, and SOA was calculated to quantify the stomatal apertures precisely. The curve of SOA overtime is drawn in Figure 6(c). From the curve of SOA over time, we found that there were distinct differences in initial aperture area of each stoma. By taking the first derivative of the curve of SOA, the stomatal area change rate curve was obtained (Figure 6(d)). The result showed that all stomata reduced size of apertures under chilling stress and closed within 10 minutes. Notably, Stoma 1, Stoma 2, and Stoma 6 closed faster than Stoma 3, Stoma 4, and Stoma 5 obviously.

### 4.3. The Correlation between SOA and Gas Exchange

**Figure 7:**
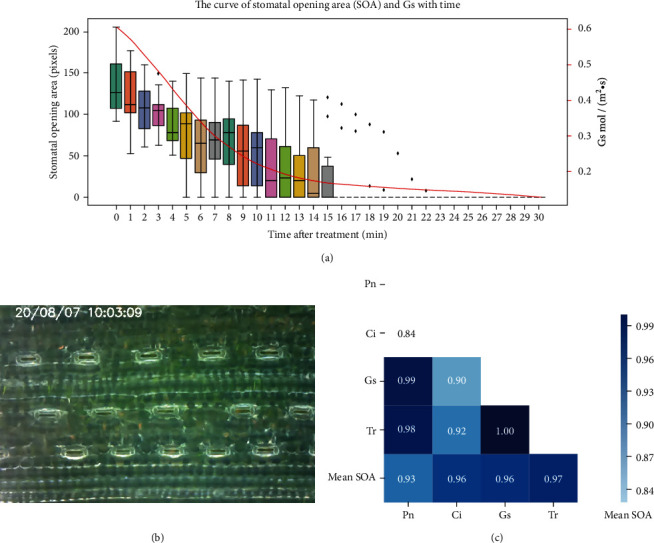
The correlation between SOA and gas exchange. (a) The curve of stomatal opening area (SOA) and Gs with time. (b) The spatial distribution of stomata. (c) The correlation heat map between mean SOA and Pn, Ci, Gs, and Tr.

We also shot the process of stomatal change in wheat under drought stress, and the relative video could be watched from Supplementary 4. The spatial distribution of stomata is shown in Figure 7(b). A box plot was used to visualize the changes of 15 stomata over time, while stomatal conductance (Gs) decreased with the similar trend (Figure 7(a)). Further, we analyzed the correlation between mean SOA and stomatal conductance (Gs), transpiration rate (Tr), intercellular CO_2_ concentration (Ci), and net photosynthetic rate (Pn) under drought stress and found significant correlations between SOA and each parameter (Figure 7(c)). The result showed that the correlation coefficients between mean SOA and Pn, Ci, Gs, and Tr are 0.93, 0.96, 0.96, and 0.97. In conclusion, SOA can be used to reflect changes in gas exchange. Notably, the results might change due to the lack of biological replicates.

## 5. Discussion

In this study, we designed a system for real-time observation and automatic analysis of wheat leaf stomata. This system can not only dynamically monitors patchy stomata in wheat leaves but also quantifies the area of each stomatal aperture automatically. Previous methods all used static pictures to analyze stomatal morphology, while our method shot the videos of stomatal movement in a real-time and noninvasive way and analyzed time-series microscope images, which could better reflect the dynamic changes of stomata. Compared with some other methods [17], we packaged this automatic analysis module into an executable software and designed a graphical user interface (GUI) in order to use this automatic analysis module smoothly. The automatic analysis module is characterized by the following features. (1) The module integrates both object tracking and semantic segmentation. (2) The module is easy to install with the aid of an executable program (EXE) (https://github.com/shem123456/Stomata-segmentation-with-GUI). (3) The functions are easily operated by a graphical user interface (GUI). (4) The results can be stored in “∗.csv” format files, and it can be exported easily for analysis using other software. In general, this automatic analysis module is very friendly to users even without any knowledge of programming. We also provided a full manual to guide users how to use it in the Supplementary Materials.

The bottleneck of the system is the robustness of semantic segmentation module. Like any other deep learning model, the robustness of this model is partially dependent on the diversity of the training dataset [27, 28]. The SBOS performs well at a variety of abiotic stresses. However, it may make a catastrophic mistake when the wheat leaves suffer from powdery mildew, because the model would mistake the bacteria for the stomata. At present, this system is only suitable for stomatal observation of wheat and barley under various abiotic stresses. The open source code can be available on the GitHub (https://github.com/shem123456/stomata-segmantic-segmentation), if researchers want to train their own semantic segmentation models depending on their specific requirements.

We successfully observed and analyzed the phenomenon of patchy stomata under chilling and drought stress. Patchy stomata are a common and characteristic phenomenon in plants, and it is significant for plant self-protection under stress [2, 29]. Studies have shown that some smaller stomata may have faster response times compared with larger stomata when plants suffer from stresses, which allowed the leaf under favorable conditions rapidly [30]. This was consistent with our result that Stoma 1, Stoma 2, and Stoma 6 closed faster than Stoma 3, Stoma 4, and Stoma 5 obviously in Experiment II. Overall, the stomatal behavior observation system (SBOS) could accurately observe the stomatal movements in different treatments. This difference in initial aperture area and stomatal closure rate may be the cause of the phenomenon of patchy stomata. Moreover, SOA is strongly correlated with stomatal conductance which is measured by the gas exchange method. Although the instrument of gas exchange can measure many parameters such as stomatal conductance (Gs), net photosynthetic rate (Pn), and intercellular CO_2_ concentration (Ci), the value will be biased due to patchy stomata, which is affected by the change of environment significantly [31]. Therefore, our system is an important complement to the gas exchange method, and it is a win-win strategy for the combination of these two methods.

Patchy stomata are thought to be helpful in the study of plant self-protection responses under stress, but there are a few reports on the study of stomata at a genetic level. Meanwhile, most of the studies on patchy stomata are focused on trees and other plants [7, 32], but there are a few studies on patchy stomata in crops. Further study of patchy stomata on crops may improve crop photosynthetic efficiency and thus increase yields [33]. Therefore, this paper chooses wheat, a common monocotyledon crop, in order to explore an intuitive and convenient method of patchy stomatal observation to better discover the formation mechanism of patchy stomata.

## Figures and Tables

**Table 1:** Test set of semantic segmentation models.

	Data augment	SE blocks	PA	CPA (background)	CPA (stomata open area)	MPA	MIoU
A	—	—	0.9633	0.9982	0.6182	0.8082	0.4962
B	+	—	0.9789	0.9977	0.6628	0.8302	0.5512
C	—	+	0.9634	0.9982	0.5392	0.7687	0.5074
D	+	+	0.9791	0.9978	0.6632	0.8305	0.5590

## Data Availability

Submission of a manuscript to *Plant Phenomics* implies that the data is freely available upon request or has deposited to an open database, like NCBI. If data are in an archive, include the accession number or a placeholder for it. Also, include any materials that must be obtained through an MTA.

## Supplementary Materials

Supplementary 1 A full manual to guide users how to use the automatic analysis module.

Supplementary 2 An installation package for the automatic analysis module.

Supplementary 3 A video of stomatal change in wheat under chilling stress.

Supplementary 4 A video of stomatal change in wheat under drought stress.

Supplementary 5 A 3D model for the platform fixed both the microscope and the plant leaf.
