# Supplementary material for: An Integrated Method for Tracking and Monitoring Stomata Dynamics from Microscope Videos
Source: Plant Phenomics. 2021 Apr 9;2021:9835961. doi: 10.34133/2021/9835961 (PMC8244544; doi:10.34133/2021/9835961)
Supplement: Supplementary 1 — A full manual to guide users how to use the automatic analysis module. [file 9835961.f1.pdf]

Automatic analysis module of SBOS

Version 1.0
December 2020
Author : Zhuangzhuang Sun
Supervisor :
Regional Technique Innovation Center for Wheat Production, Ministry of Agriculture, Key Laboratory of Crop Physiology and Ecology in Southern China, Ministry of Agriculture, Nanjing Agricultural University, Nanjing, China

This package includes:
(1) stomata.exe
(2) model.h5
(3) test.mp4
(4) intro.mp4

This software can run on Windows 10 with a 64-bit Operating System, x64-based processor.

Table of contents :
1. Download files
2. Use the Graphical User Interface (GUI)
2.1 Open “stomata.exe”
2.2 Select the video file
2.3 Set up the parameter
2.4 Enter the path and name of results
2.5 Select the stomata of interest

1. Download files

Go to the address : https://pan.baidu.com/download
Click on ‘Download’ for Baidu cloud disk

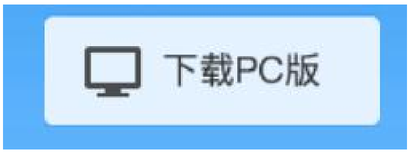

Go to the link: https://pan.baidu.com/s/1lU3xw_g_aWGDYoiuQFbkeQ
Extraction code: SKV7
(If this link doesn’t work, you can email me: 2020201018@stu.njau.edu.cn)

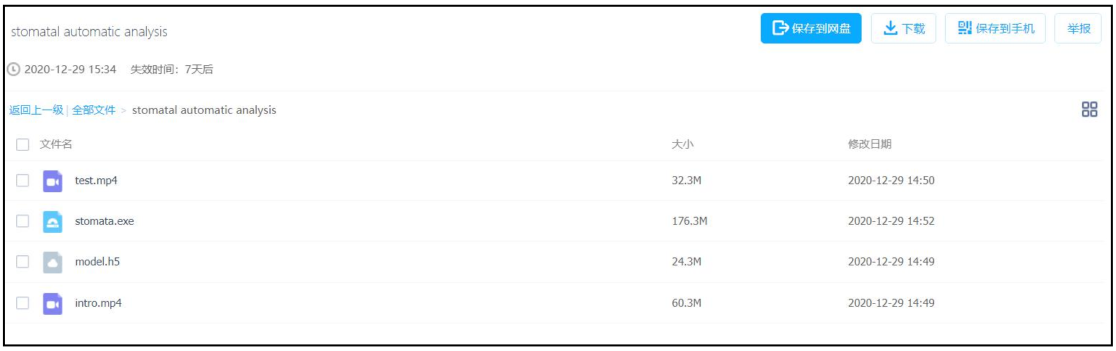

Download all files to your local computer.

2. Use the Graphical User Interface (GUI)

2.1 open "stomata.exe"

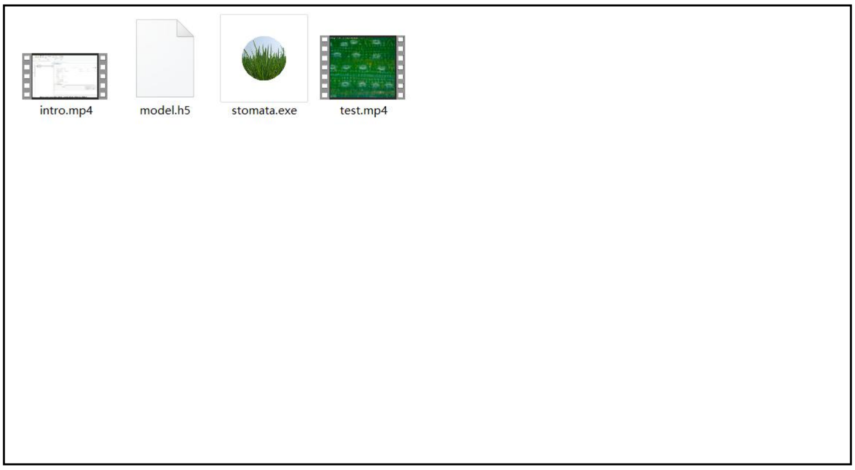

Click on "stomata.exe", and you will see the GUI.

2.2 Select the video file

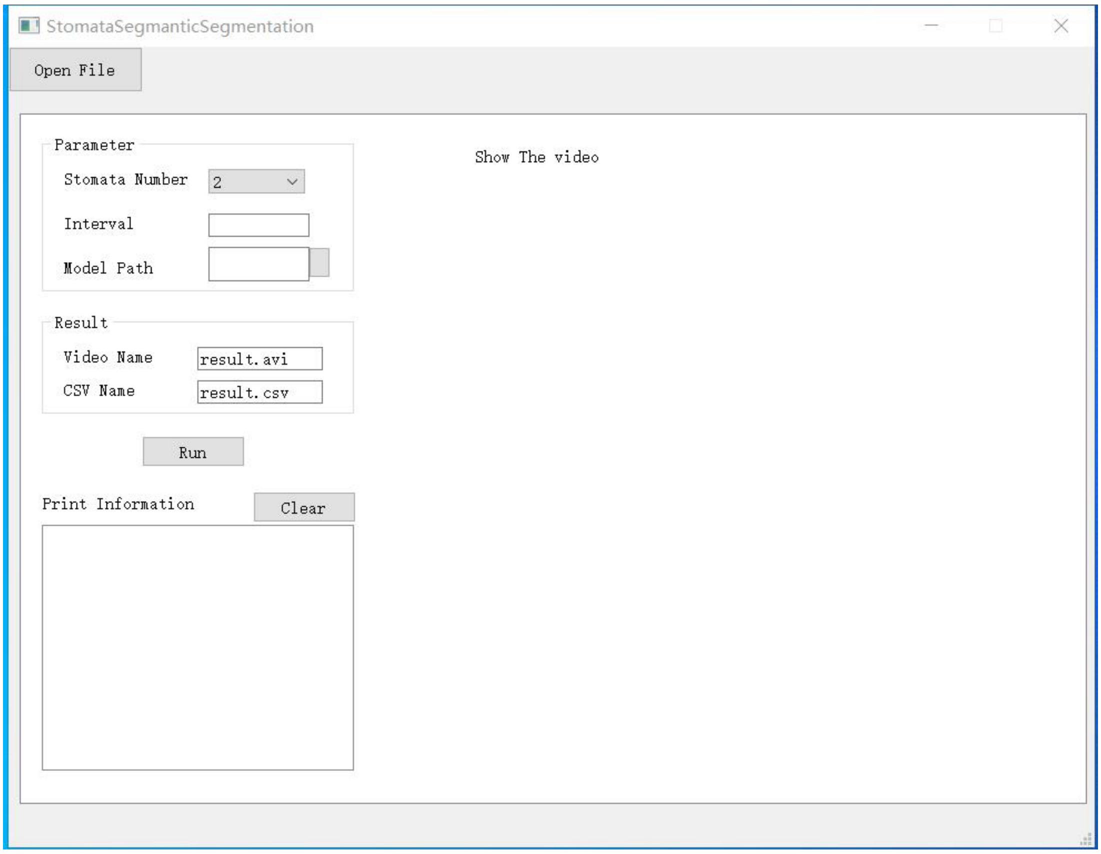

Click on the icon 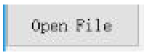

at the top left corner and choose the video you want to test.

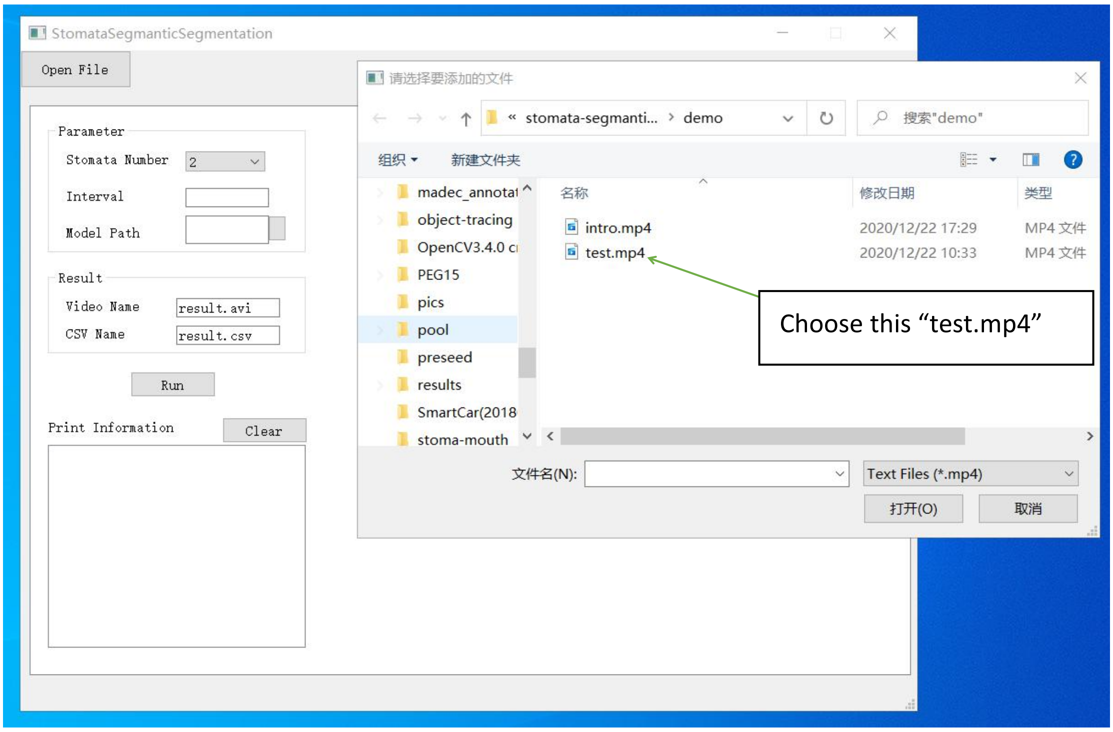

2.3 Set up the parameter

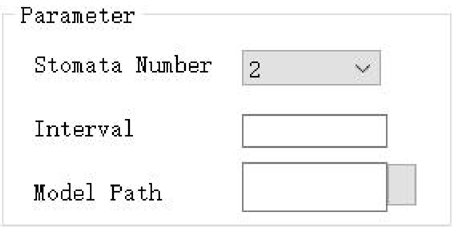

Stomata Number 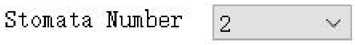

: the range is 2 to 10, and it represents the number of stoma you want to choose. Because the first box is used to select the time and date area, its minimun value is 2.

Interval: it represents semantic segmentation every n frames.

Model Path: select a semantic segmentation model. Here, we provide a model (model.h5) for testing.

2.4 Enter the path and name of results

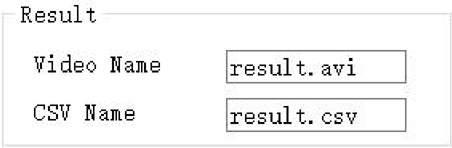

Modify the path and name of the output, and the default are “result.avi” and “result.csv”.

2.5 Select the stomata of interest

Click “Run” 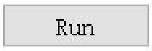

, and you can see the first frame of the video.

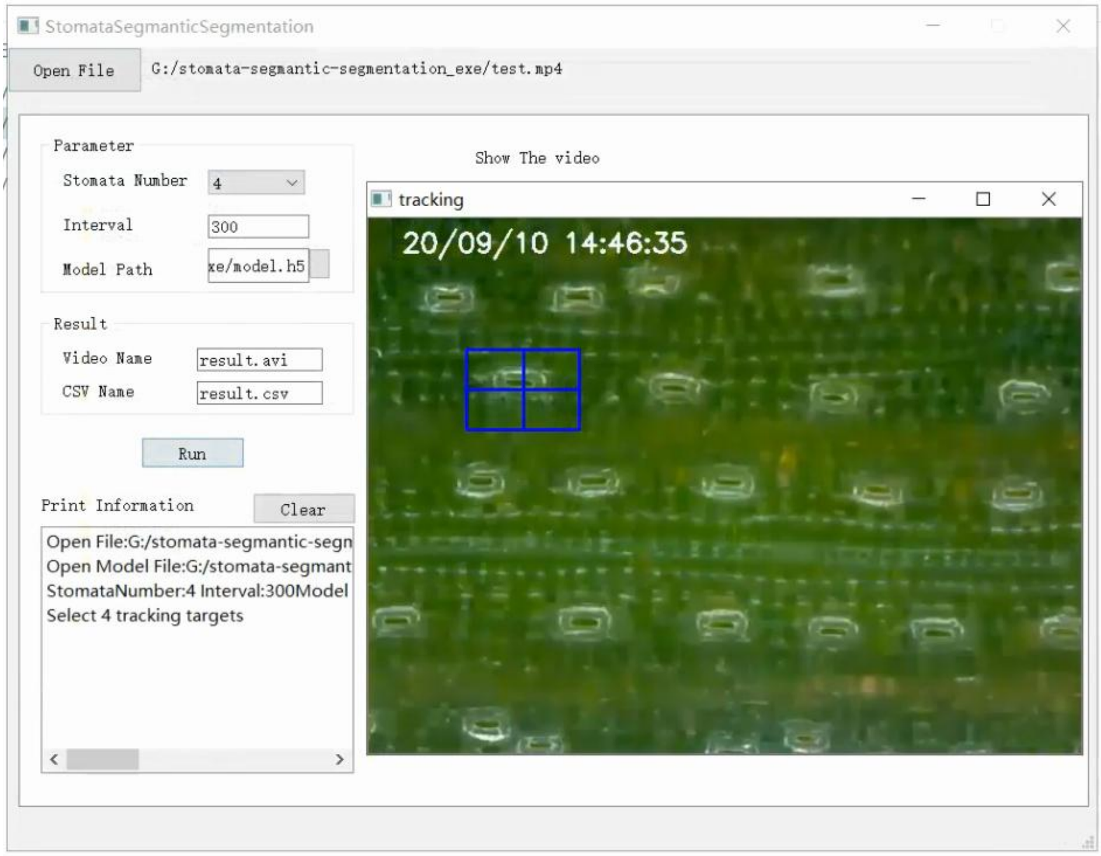

The first box must select the time and date area, and the other boxes choose the stomata of interest. After drawing a rectangle, press "Enter" on the keyboard to confirm.

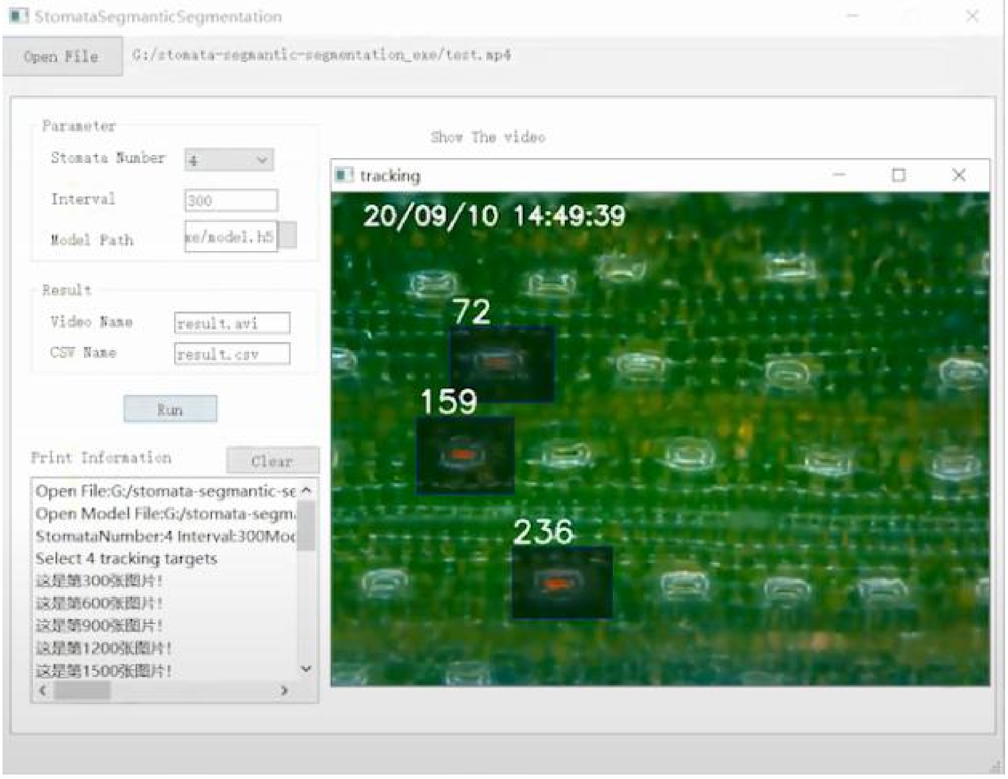

When finished, the ".csv" file and the video file will be saved in the local folder.

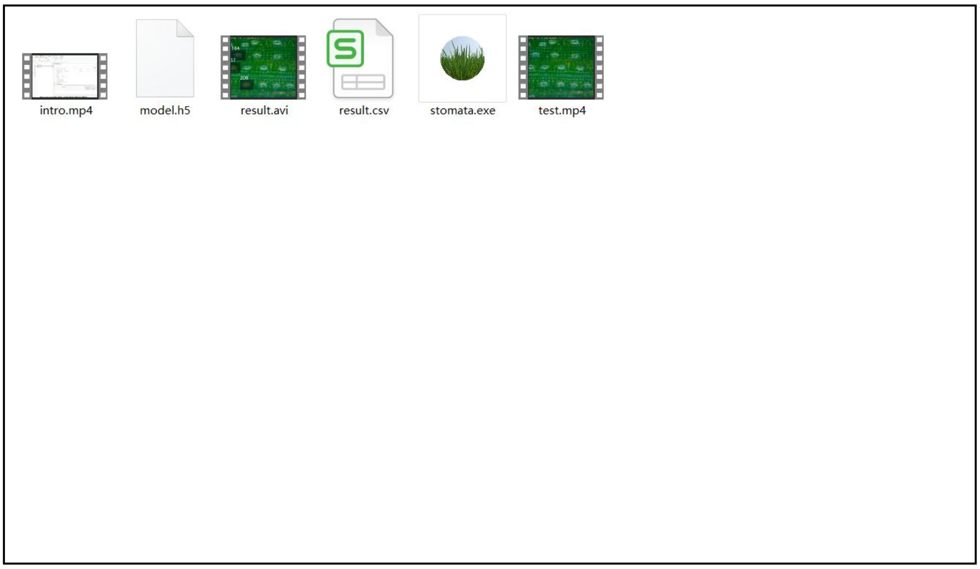
intro.mp4
model.h5
result.avi
result.csv
stomata.exe
test.mp4
